# Editorial: Preparation of organic shell nanoparticles and their application in the synthesis of organic compounds

**DOI:** 10.3389/fchem.2023.1313772

**Published:** 2023-11-16

**Authors:** Leila Zare Fekri

**Affiliations:** Department of Chemistry, Payame Noor University, Tehran, Iran

**Keywords:** organic shell nanoparticles, quinazolinone derivatives, MOFs, lignin-based spherical particles, antibacterial properties

Shiri et al., reported a one-pot multicomponent reaction of benzaldehydes, dimedone, and 1H-1,2,4-triazol-3-amine for the efficient synthesis of quinazolinone derivatives under green conditions using MIL-101(Cr). In recent years, tremendous development has been achieved in the field of MOFs (metal−organic frameworks) architectures as a novel class of porous materials. The MOFs incorporate the advantages of sustainable synthesis with heterogeneous catalysis, which indeed make easier workup procedure, and are more applicable in industries and academics. Due to global energy and environmental problems, the finding of unique catalysts in a simple way with high reusability has become increasingly crucial in managing the associated challenges. In this work, the catalyst is reusable for eight runs. On the other hands, the reaction was carried out at room temperature that is based on green chemistry rules because the process is energy-saving.

MIL-101(Cr) was synthesized and characterized by different analytical methods such as FT-IR, SEM, and EDX.

**SCHEME 1:**
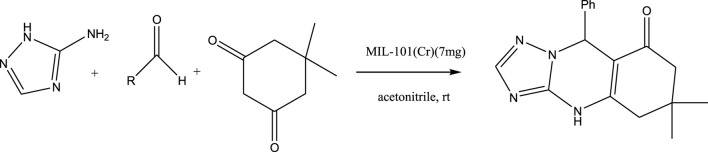
Synthesis of quinazolinones using MIL-101 (Cr).

For the synthesis of catalyst, first, a mixture of Cr (NO_3_)_3_·9H_2_O (5.4 g) and terephthalic acid (1.5 g) was added to deionized water (45 mL) and hydrofluoric acid (0.6 mL, 5 mol L^−1^) in a Teflon-lined stainless-steel autoclave. After sonication for 10 min, the mixture was heated in an oven at 220°C for 9 h. In continuation, the mixture was cooled down to r. t. and then the mixture was filtered and washed several times with hot water and hot DMF. The resulting MIL-101(Cr) catalyst was dried and purified further with hot filtration in DMF at 120°C for 12 h. The solid was washed several times with hot DMF and hot ethanol. Finally, the solid was filtered and dried at 80°C for 6 h. This catalyst after characterization was used for the multicomponent synthesis of quinazolinones (Scheme 1). The aldehydes with various substituents underwent this methodology. The results are summarized in Table 1.

**TABLE 1:** The scope and generality of synthesis of quinazolinones.

R	Time (min)	Yield (%)^a^
Ph	30	94
4-Me C_6_H_4_	30	91
4-OCH_3_ C_6_H_4_	30	89
4-F C_6_H_4_	30	90
4-Cl C_6_H_4_	30	92
4-NO_2_ C_6_H_4_	30	95
2-Me C_6_H_4_	30	88
3,4-diOMe C_6_H_3_	30	85
4-OH C_6_H_4_	30	87
4-N,N-diMeN	30	84
2-Cl C_6_H_4_	30	89
α-Naphthyl	30	81
3-NO_2_ C_6_H_4_	30	90

^a^ Isolated yields.

Shiri et al., carried out the high scaled reaction of benzaldehyde, dimedone and 1H-1,2,4-triazol-3-amine in this avenue. They reported in 30 min, 91% of product was gained per 10 mmol of substrate.

In order to reuse MIL-101(Cr) as a novel catalyst, the mixture of reaction was centrifuged to separate the MIL-101(Cr) catalyst, and then the MIL-101(Cr) catalyst was washed several times with DCM. After drying, the MIL-101(Cr) catalyst was utilized for the next run. To our delight, the MIL-101(Cr) catalyst could be used for eight runs.

Shiri et al., have stated that the first run of using the catalyst has a 94% yield, but in the next runs, the yields decrease, and the eighth run has an efficiency of 84%, which presents a significant drop. It seems that the recycling properties of the catalyst are not as beneficial as mentioned by Shiri and his colleagues in the article. In any case, in order to write an editorial, I am obliged to express the things written in Shiri s article as mentioned.

The outstanding benefits of this methodology are the availability of substrates, using green conditions, excellent functional group compatibility, and reusability of catalysts, therefore providing easy access to a range of products of interest in organic and medicinal chemistry.

Stanisz et al. reported synthesis of lignin-based spherical particles (Lig-IL) with the use of 1-(propoxymethyl)-1H-imidazolium hydrogen sulfate were prepared in different biopolymer and ionic liquid (IL) weight ratios.

**SCHEME 2:**
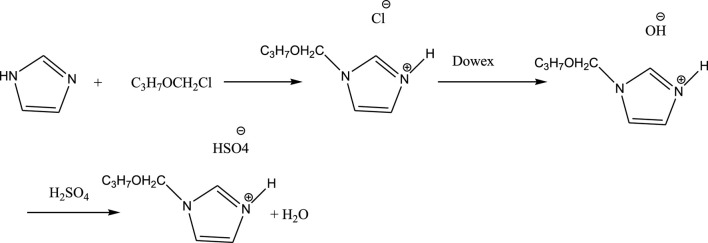
Synthesis of acidic imidazolium ionic liquid.

The synthesis of acidic imidazolium ionic liquid consisted of three steps. In the first step, imidazolium chloride was obtained by a reaction of imidazole with chloromethyl propyl ether. Chloromethyl propyl ether was obtained by passing HCl gas through a mixture of formaldehyde and the appropriate alcohol. In the second step, chloride anions in imidazolium chloride were exchanged for hydroxide anions, with the use of an ion-exchange resin (Dowex Monosphere 550 A UPW OH form resin). In the third step, the obtained imidazolium hydroxide was immediately subjected to a reaction with sulfuric acid and the corresponding imidazolium hydrogen sulfate was obtained. Water was evaporated in a vacuum. 1-(propoxymethyl)-1H-imidazolium hydrogen sulfate was synthesized (Scheme 2).

For the preparation of Lig-IL, kraft lignin was combined with 1-(propoxymethyl)-1H-imidazolium hydrogen sulfate for the preparation of biopolymer-based spherical particles with the use of the soft templating method. The lignin-based particles were obtained with the use of precursors in different weight ratios namely 1:1, 4:3, 2:1, 4:1, and 10:1.

Produced lignin-based spherical particles were used for evaluation of their antibacterial properties. Particles were tested against *Staphylococcus aureus* (*S. aureus*), a Gram-positive bacterium, and *Escherichia coli* (*E. coli*), a Gram-negative one. It was observed, that only the material with the highest addition of IL showed the antibacterial properties against both strains. A reduction of 50% in the number of microorganisms was observed for particles with the addition of hydrogen sulfate ionic liquid in a 1:1 ratio after 1 h. However, all prepared materials exhibited the antibacterial activity against a Gram-positive bacterium.

**SCHEME 3:**
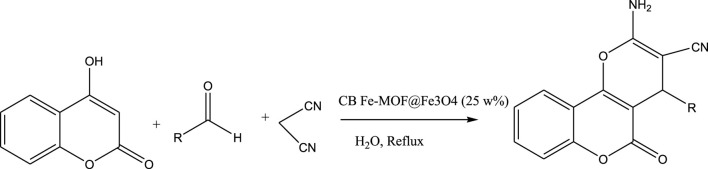
Multicomponent synthesis of 3,4-dihydropyrano[3,2-c]chromene.

Sheikhhosseini and Yahyazadehfar reported the synthesis of recyclable heterogeneous cluster bud Fe-MOF@Fe_3_O_4_ “nanoflower” composite (CB Fe-MOF@Fe3O4 NFC). The CB Fe-MOF@Fe3O4 NFC was used in the synthesis of dihydropyrano[3, 2-c]chromene derivatives via a three-component reaction of 4-hydroxycoumarin, malononitrile, and a wide range of aromatic aldehyde compounds (Scheme 3).

For the synthesis of catalyst, initially, nano Fe_3_O_4_ was prepared using mixing of iron (III) nitrate and iron (II) Chloride. Then, 8-hydroxyquinoline sulfate monohydrate-linker reacted with iron nitrate to synthesize Fe-MOF. In the final step, Fe_3_O_4_ was added to Fe-MOF to preparation of CB Fe-MOF@Fe_3_O_4_.

To optimize the catalytic conditions, the reaction of malononitrile, 4-hydroxycoumarin, and 3-nitrobenzaldehyde was chosen as a model reaction. Initially, the reaction was carried out using various amoun of Fe-MOF@Fe _3_ O _4_ (5%, 10%, 15%, 20%, 25% and 30%) in refluxing water as solvent. 25% of catalyst was the best. Then, the model reaction was carried out with optimized amount of catalyst in the presence of different solvents as acetonitrile, MeOH: H_2_O, EtOH:H_2_O, MeOH, EtOH and under solvent-free condition. The best result for the reaction to progress was attained with the use of 0.25 w% of CB Fe-MOF@Fe _3_ O _4_ NFC in H _2_ O under reflux conditions. In comparison of this method, the model reaction was carried out in the presence of Fe_3_O_4_ 25% in refluxing water. But the results showed that Fe-MOF@Fe _3_ O _4_ is a better choice as catalyst.

Under optimal conditions, various aromatic aldehydes were added in the presence of CB Fe-MOF@Fe _3_ O _4_ NFC to test the applicability of this technique (TaThe synthesized catalyst was used for the multicomponent reaction of malononitrile, aldehydes and 4-hydroxycoumarine and synthesis of 3,4-dihydropyrano[3,2-c]chromenes (Scheme 3). The results were summarized in Table 2.

**TABLE 2:** The scope of synthesis of 3,4-dihydropyrano[3,2-c]chromenes.

R	Time (min)	Yield (%)^a^
3-Cl C_6_H_4_	5	95
3,4,5-triOMe C_6_H_2_	29	93
2-OMe C_6_H_4_	5	92
4-OH C_6_H_4_	7	91
4-NO_2_ C_6_H_4_	10	98
3-NO_2_ C_6_H_4_	3	96
4-Cl C_6_H_4_	6	97
4-OMe C_6_H_4_	5	95
2-Me C_6_H_4_	6	81
Ph	2	97
2,4-diOMe C_6_H_3_	6	98
2-Me C_6_H_4_	4	95

^a^ Isolated yields.

After completion of the reaction, the mixture was diluted with ethanol and the catalyst was separated using an external magnet, followed by washing with hot ethanol and drying in an oven. According to our results, the catalyst was reusable five successive times with no significant loss in reaction yield.

Optimized reaction conditions had several advantages, including the use of water as a green solvent, environmental compatibility, and simple work-up, reusability of the catalyst, low catalyst loading, faster reaction time, and higher yields.

**FIGURE 1:**
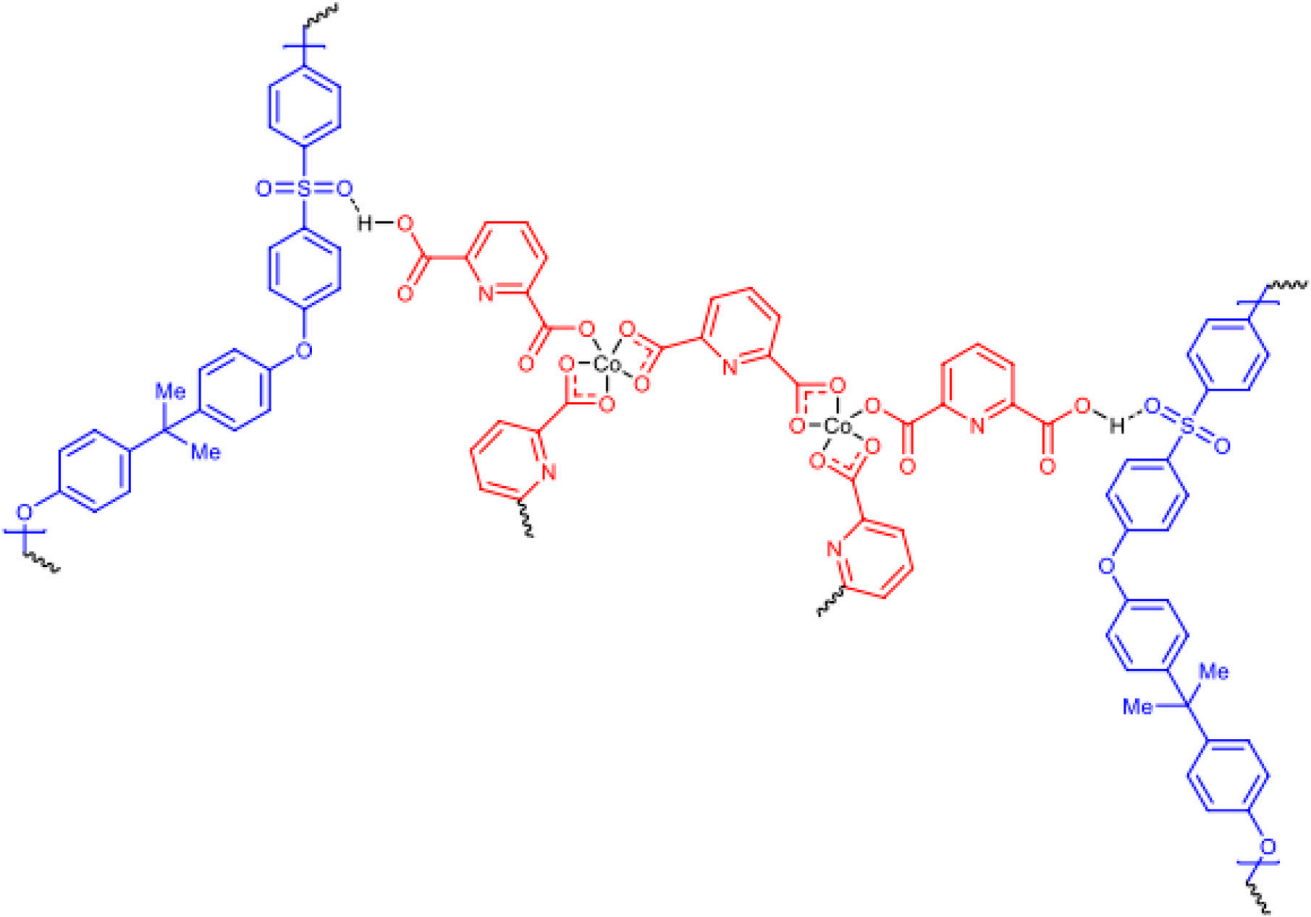
The structure of cobalt composite immobilized on polysulfone fibrous network nanoparticles (CCPSF NPs).

Mustafa et al., reported synthesis of cobalt composite immobilized on polysulfone fibrous network nanoparticles (CCPSF NPs) (Figure 1) and its application for the oxidation of the primary and secondary alcohols to aldehyde and ketone under solvent-free microwave-assisted conditions Ramirez-Colone et al. In addition to oxidation properties, the anticancer activity of the synthesized CCPSF NPs in breast cancer was evaluated by the MTT method, and significant results were obtained. The IC 50 values of CCPSF NPs at 24 and 48 h, 149.0312 and 110.3137 mg/mL, respectively, were obtained. The cell proliferation and viability were then controlled at a concentration of 200 mg/mL at 24 and 48 h, and 38.9% and 28.8%, respectively, were observed. Based on the obtained results, it can be concluded that the effect of CCPSF NPs on MCF-7 breast cancer cells depends on concentration and time, and the impact increases with increasing concentration and time. The anticancer activity of CCPSF NPs can be attributed to the presence of polysulfone (with high biological properties) and cobalt in their structure, as well as its high specific surface area, which was created as a result of the appropriate synthesis method.

For the preparation of catalyst, at first, amixture of Co (NO_3_)_2_ and pyridine-2,6 dicarboxylic acid was stirred to synthesize cobalt composte. With mixing of polysulfone powder and cobalt composite, cobalt composite immobilized on polysulfone fibrous network nanoparticles were prepared.

**SCHEME 4:**
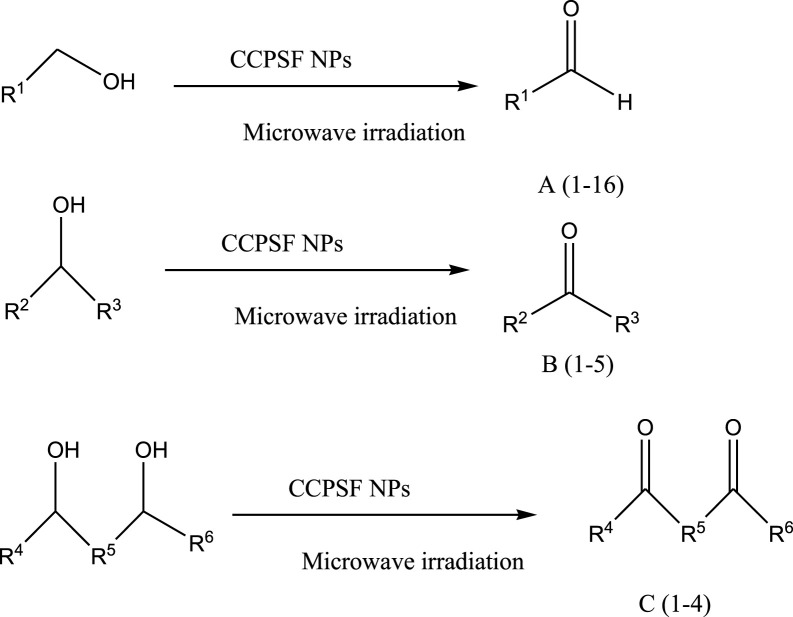
Oxidation of alcohols using cobalt composite immobilized on polysulfone fibrous network nanoparticles.

The prepared catalyst was used for oxidation of alcohols under microwave condition (Scheme 4). The results were summarized in Table 3.

**TABLE 3:** The scope of oxidation of alcohols using cobalt composite immobilized on polysulfone fibrous network nanoparticles.

Product	Compound	Time (min)	Yield (%)
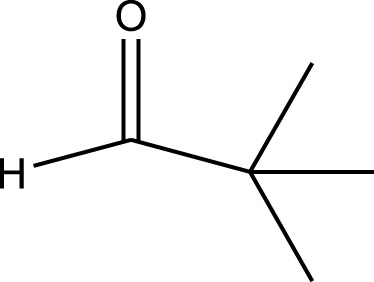	A1	2	91
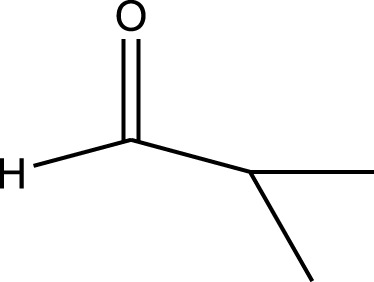	A2	3	92
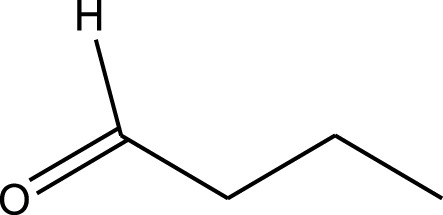	A3	2	90
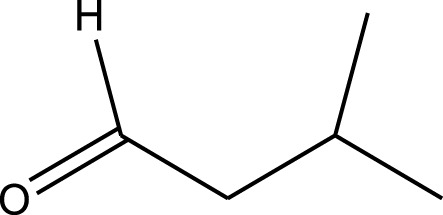	A4	2	91
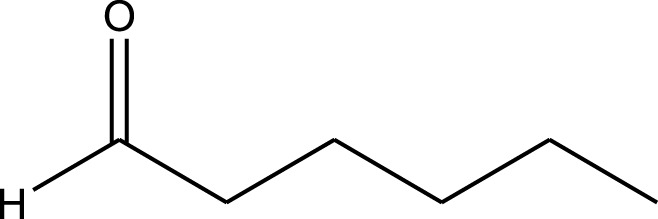	A5	2	91
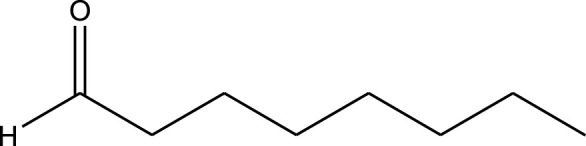	A6	2	95
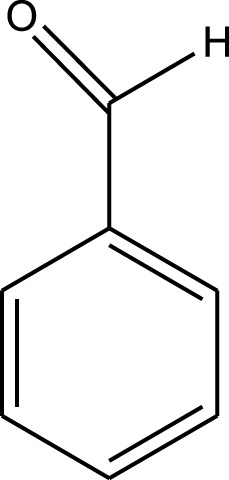	A7	2	100
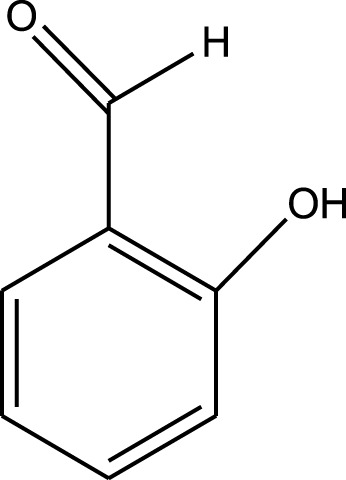	A8	2	100
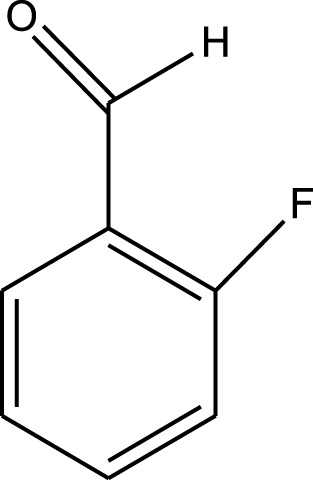	A9	2	93
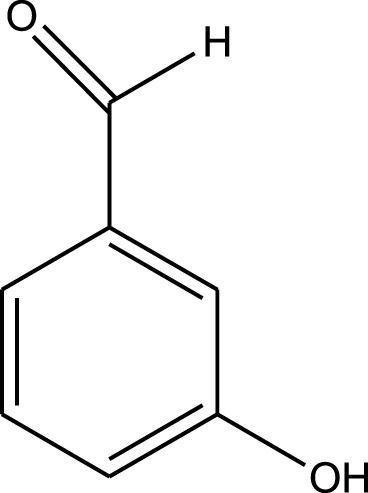	A10	2	98
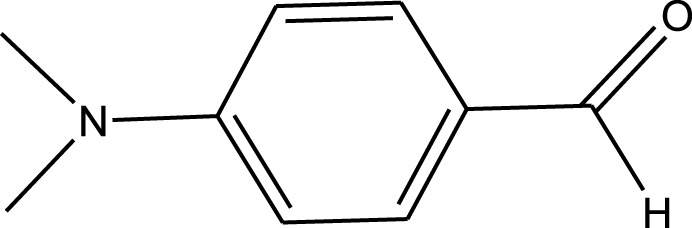	A11	2	97
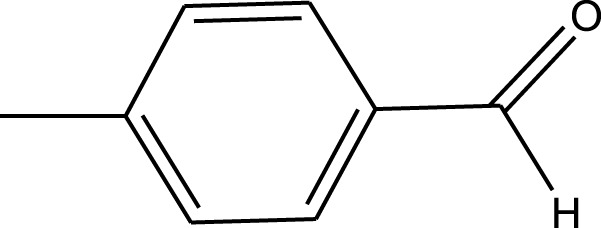	A12	2	100
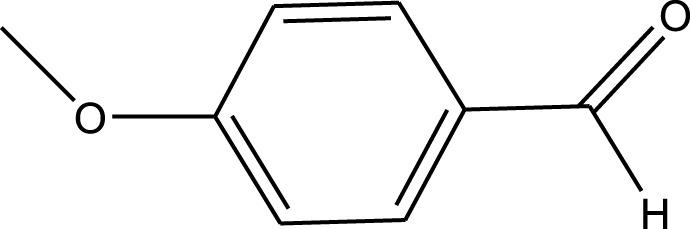	A13	2	100
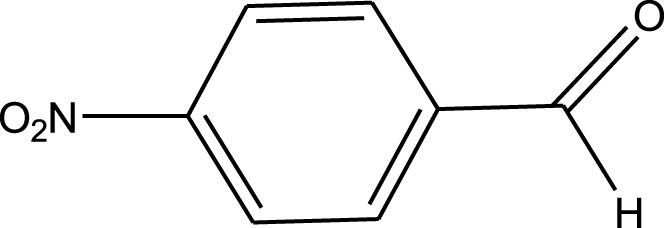	A14	2	96
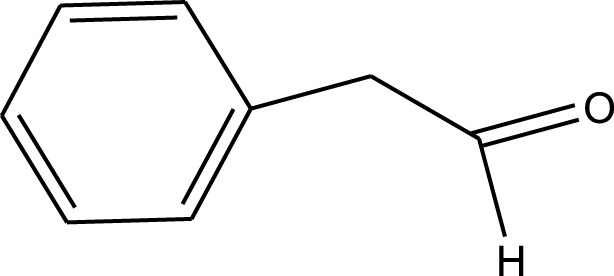	A15	2	95
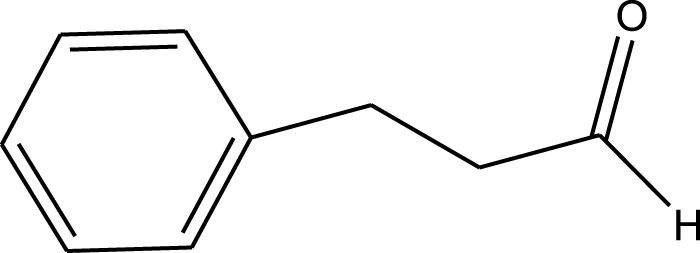	A16	3	92
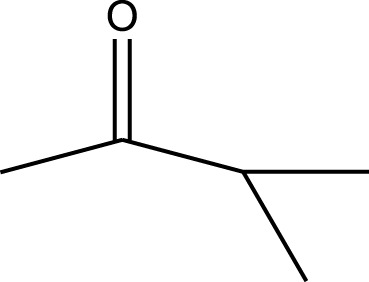	B1	3	91
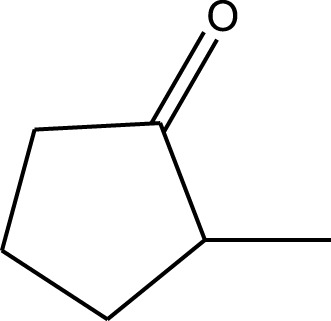	B2	4	92
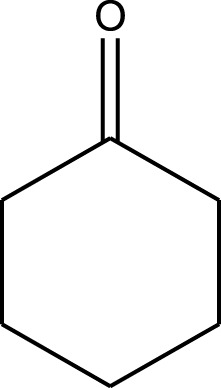	B3	3	93
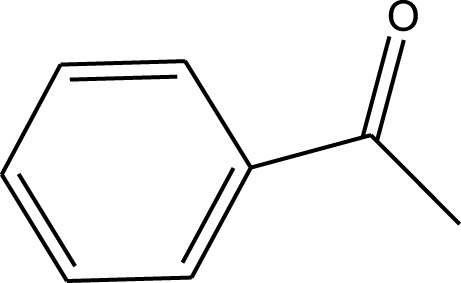	B4	2	100
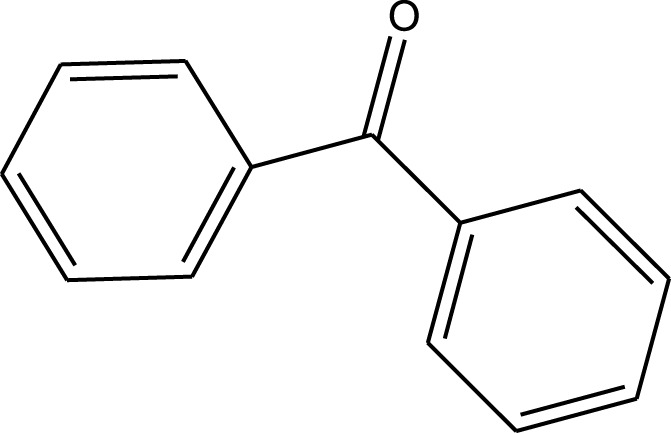	B5	2	97
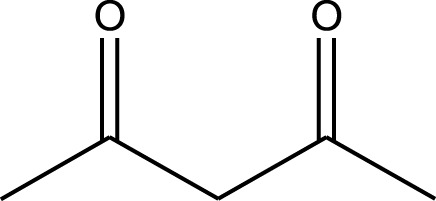	C1	3	93
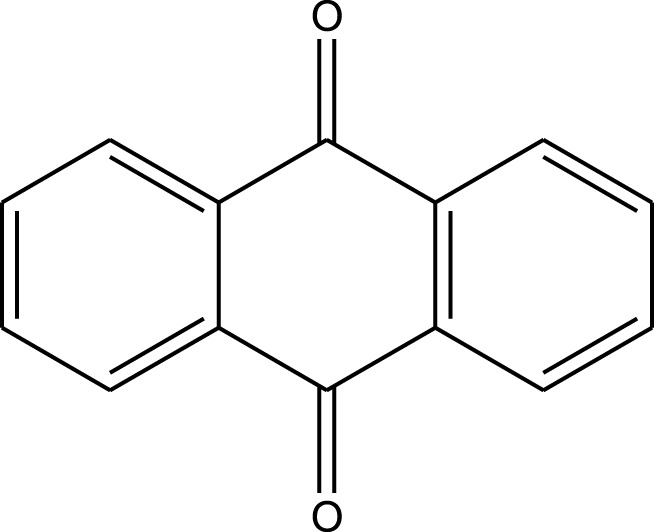	C2	2	100
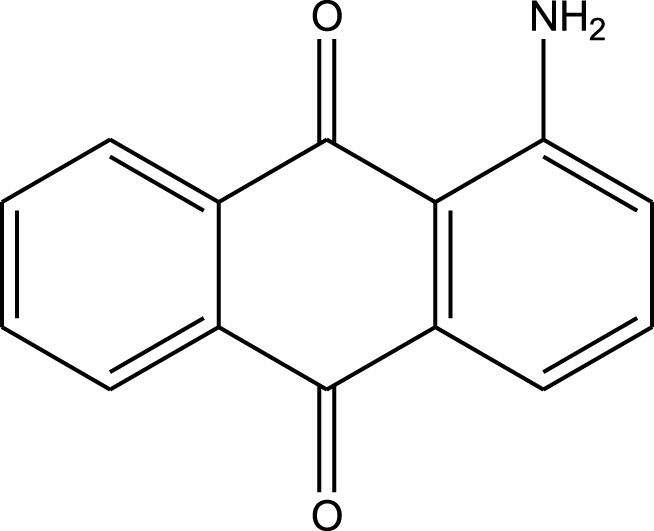	C3	3	95
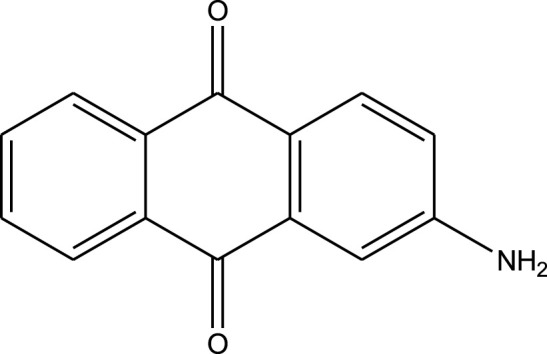	C4	4	93

